# Role of Fibronectin-1 polymorphism genes with the pathogenesis of intraventricular hemorrhage in preterm infants

**DOI:** 10.1007/s00381-020-04598-3

**Published:** 2020-04-13

**Authors:** Dawid Szpecht, Salwan R. Al-Saad, Lukasz M. Karbowski, Katarzyna Kosik, Grażyna Kurzawińska, Marta Szymankiewicz, Krzysztof Drews, Agnieszka Seremak-Mrozikiewicz

**Affiliations:** 1 Chair and Department of Neonatology, Poznan University of Medical Sciences, Poznan, Poland; 2 Poznan University of Medical Sciences, Poznan, Poland; 3 Department of Perinatology and Women’s Diseases, Poznan University of Medical Sciences, Poznan, Poland

**Keywords:** Fibronectin-1, Polymorphism genes, Intraventricular hemorrhage, Preterm infants

## Abstract

**Background/introduction:**

Intraventricular hemorrhage (IVH) is a dangerous complication facing a significant proportion of preterm infants. It is multifactorial in nature, and an observed fibronectin deficiency in the germinal matrix basal lamina is among the most prominent factors that influence such rupture. Better understanding of the FN1 gene polymorphisms and their role in IVH may further clarify the presence of a genetic susceptibility of certain babies to this complication. The aim of this study was to assess if 5 single nucleotide polymorphisms of the fibronectin gene may be linked to an increased incidence of IVH.

**Material and methods:**

The study included 108 infants born between 24 and 32 weeks of gestation. IVH was diagnosed using cranial ultrasound performed on the 1st,3rd, and 7th day after birth and classified according to Papile et al. IVH classification. The 5 FN1 gene polymorphisms assessed in the study were the following: *rs3796123*; *rs1968510*; *rs10202709*; *rs6725958*; and *rs35343655.*

**Results:**

IVH developed in 51 (47.2%) out of the 108 preterm infants. This includes, 18 (35.3%) with stage I IVH, 19 (37.3%) with stage II, 11 (21.6%) with stage III, and 3 (5.9%) with stage IV IVH. Incidence of IVH was higher in infants with lower APGAR scores, low gestational age, and low birthweight. Analysis showed that IVH stage II to IV was approximately seven times more likely to occur in infants with the genotype TT FN1 *rs10202709* (OR 7237 (1046–79.59; *p* = 0,044)). No other significant association was found with the rest of the polymorphisms.

**Conclusion:**

The results of our study indicate a sevenfold increased genetic susceptibility to IVH in preterm infants with the TT FN1 rs10202709 gene polymorphism. The fibronectin gene polymorphism may therefore be of crucial importance as a genetic risk factor for IVH in preterm infants. Further studies are warranted.

**Electronic supplementary material:**

The online version of this article (10.1007/s00381-020-04598-3) contains supplementary material, which is available to authorized users.

## Introduction

Intraventricular hemorrhage (IVH) is one of the most severe complications of preterm birth. In some cases, it may lead to death, and on the other hand a significant proportion of preterm infants who do survive following this complication suffer either disabling neurological consequences or neuropsychological problems [1, 2]. Although neonatal intensive care has been improved all over the world, IVH occurs in about 20% of infants born before 32nd week of gestation [3]. Mortality rates are 4% for grade I, 10% for grade II, 18% for grade III, and 40% for grade IV, with outcomes for IVH grade I or II being similar to infants without IVH [4].

Presently, the etiopathogenesis of IVH seems multifactorial and not entirely clear. Some major risk factors include the following: body weight at birth (less than 1500 g), gestational age (below 32 weeks), absence of prenatal steroid therapy in women at risk of premature delivery, early clamping of the umbilical cord, among others [5]. Furthermore, in IVH pathogenesis are involved: irregularities in cerebral blood flow, coagulation and platelet abnormalities which further accentuate the bleeding, and very critical in preterm infants in the integrity of the germinal matrix [6]. Genetic factors participating in the development of IVH are still being analyzed. Analysis of the role of genetic factors in the pathophysiology of IVH will make it possible to determine the group of newborns who are specifically at risk of developing IVH in the perinatal period. The following genetic factors that may predispose to IVH have been analyzed so far: polymorphisms of genes coding for proinflammatory cytokines [7, 8], coagulation pathway [9–11] regulation of systemic blood pressure and cerebral blood flows [12], as well as structural components of the germinal matrix [13, 14]. There is still not enough definitive information about the connection between the increased risk of IVH and the molecular structure of the vasculature in the germinal matrix.

In developing fetuses, the germinal matrix constitutes a temporary layer on the subependymal walls of the brain lateral ventricles present at 8 weeks of gestation until roughly 36 weeks when it involutes [15], with a maximum volume reached between 23 and 28 weeks of gestation [16]. Having a rich vasculature due to high nutritional requirement by differentiating glial/neuronal cells [17], the germinal matrix is considered a highly sensitive portion of the brain and usually the starting site of IVH in preterm infants.

Of the components making up the BBB in the vasculature of the germinal matrix [18, 19], the basal lamina is of particular interest in this discussion of IVH. The basal lamina in the vascular system is built of fibronectin, collagen, laminin, perlecan, and heparan sulfate proteoglycan [20, 21]. Previous studies have shown that heterozygous mutations in the type IV procollagen gene, COL4A1 (4582–4586dupCCCATG insertion), are associated with fetal intraventricular hemorrhage [13].

There is also evidence of decreased expression of fibronectin in the germinal matrix basal lamina compared with basal lamina of vessels in other parts of the central nervous system [22]. Since fibronectin takes part in regulating the extracellular matrix stability [23], the decreased expression in the germinal matrix may further weaken the vasculature. These reports create a basis for analyzing fibronectin gene polymorphisms and determine its meaning in occurrence of IVH.

## Material and methods

### Study population

The study includes a population of 108 infants born from 24 + 0 to 32 + 0 weeks of gestation, hospitalized at the Department of Neonatology (III level hospital) of Poznan University of Medical Sciences.

The study did not include neonates born before 24 + 0 and after 32 + 0 weeks of pregnancy, as well as those born from multiple pregnancies, from pregnancies complicated by death of one of the fetuses, chromosomal abnormalities, TORCH (toxoplasmosis, other, rubella, cytomegalovirus, herpes) complex inflammation, or inherited errors of metabolism. Infants without antenatal steroid therapy (AST) were also excluded from the study.

### Clinical features

The following factors that may be associated with the development of IVH were studied: gender, gestational age (GA; weeks), and birth weight (BW, grams); mode of delivery (vaginal birth vs cesarean section); APGAR score; pH < 7.0 and blood base excess (BE) in cord blood; place of birth (inborn/outborn); and intrauterine infection (defined as positive culture in sterile originally accompanied by clinical symptoms or pneumonia developed in 48 h after the birth).

### IVH diagnosis

IVH was diagnosed with the use of cranial ultrasound (10 MHZ transducer, Prosoundα7 Premier, Aloka). Routine cranial ultrasound examinations were performed on 1st, 3rd, and 7th day after birth, in accordance with local standards and recommendations in infants born earlier than 32 weeks gestation. The maximal degree of IVH was confirmed by cranial ultrasound on the 7th day of life. The classification of intraventricular bleeding was based on the Papile et al. IVH classification [24].

### Studied polymorphisms

The criteria for selection of candidate genes in the present study were their potential involvement in the pathogenesis of IVH [25, 26].

We studied 5 single nucleotide polymorphisms of fibronectin gene which are the following: *rs3796123*; *rs1968510*; *rs10202709*; *rs6725958*; and *rs35343655.* Due to lack of available primer sequences in previous publications of the *rs3796123* polymorphism, the primer for this polymorphism was constructed using the software Primer3web (version 4.1.0) [27]. Both alleles and genotypes were included in the assessment in order to more thoroughly analyze the distribution of correlated factors with the syndrome [28].

A sample of blood was taken directly after the delivery and banked. Genomic DNA was extracted from blood leukocytes using QIAamp DNA Blood Mini Kit (QIAGEN Inc; Germany). Genotyping was performed using polymerase chain reaction (PCR) procedures. Description of the studied polymorphisms genes is shown in Table 1.

**Table 1:** Description of studied polymorphisms

Polymorphism	Sequence of primers	Literature	Products
*rs3796123*	5’-ACC AAT gCCAgg ATT CAg Ag-3’ 5’-CCC AAC TTA ggCATgAgA gC-3’	Untergasser et al. (2012)	AluI
*rs1968510*	5’-gTT TgTTgTgTCAgTgTA gTA-3’ 5’-TgC ATT AgCgTTATggCCATg-3’	Avila et al. (1999)	TaqI
*rs10202709*	5’-CAg TCC CAg ATC ATggAg TCT-3’ 5’-gTA CCA TgT TAC TTgTgg AAT AgA g-3’	Murat et al. (2015)	HindIII
*rs6725958*	5’-CTC AggACTTggATggTgTAg A-3’ 5’-TCA TTT CCC AAT AAA AgT ACA CTg-3’	Murat et al. (2015)	HaeIII
*rs35343655*	5’-ACT gAAgTg CTC gggATg AT-3’ 5’-CAg gAACgA AAT gTTggA Tg-3’	Murat et al. (2015)	MspI

### Statistical analysis

The results are presented as a percentage for categorical variables, or median (range) for non-normally distributed continuous variables as tested by the Shapiro–Wilk test. A *p* value of less than 0.05 was considered significant. The Fisher exact probability test, the chi-square test, Fisher Freeman Halton, and Chi-squared test with Yates correction were used to evaluate the association between IVH and categorical variables. Differences in non-normally distributed continuous variables were compared by the U Mann–Whitney test. Statistical analysis was performed using CytelStudio version 10.0, created January 16, 2013 (CytelStudio Software Corporation, Cambridge, Massachusetts, United States), and Statistica version 10, 2011 (Stat Soft, Inc., Tulsa, Oklahoma, United States).

## Results

Table 2 shows the demographic and clinical characteristics of enrolled infants. In our study population, 51 (47.2%) infants developed IVH. The infants were diagnosed according to stages of IVH; 18 (35.3%) newborns were diagnosed with IVH stage I, 19 (37.3%) with stage II, 11 (21.6%) with stage III, and 3 (5.9%) with stage IV IVH.

**Table 2:** Demographic and clinical characteristics of enrolled infants

	Group without IVH and I *N* = 75 (%)	Group with IVH stage II- IV *N* = 33 (%)	*P* value
Gender			0.08666^a^
Male	39 (52%)	23 (69.7%)	
Female	36 (48%)	10 (30.3%)	
Gestational age (week)			0.00003^a^
24 + 0–28 + 6	31 (41.3%)	28 (84.8%)	
29 + 0–32 + 0	44 (68.7%)	5 (15.2%)	
Birth weight (gram)			0.00007^a^
< 750	5 (6.70%)	9 (27.3%)	
750–1000	17 (22.7%)	15 (45.4%)	
> 1000	53 (70.6%)	9 (27.3%)	
Apgar score (Median and range)			
1st minute	6 (1–10)	4 (1–10)	0.000127^d^
5th minute	8 (2–10)	7 (1–9)	0.000240^d^
Mode of delivery			0.1252^a^
Vaginal	29 (38.7%)	18 (54.5%)	
Cesarean section	46 (61.3%)	15 (45.5%)	
pH	7.33 (7.02–7.53)	7.29 (6.98–7.51)	0.12624^d^
BE	− 2.65 (− 14.7–+ 10.6)	−2.8 (− 10.7–+ 0.8)	0.538729^d^
Intrauterine infection			0.009^a^
YES	39 (52%)	26 (78.8%)	
NO	36 (48%)	7 (21.2%)	
Inborn	68 (90.7%)	28 (84.8%)	0.57964^c^
Outborn	7 (9.3%)	5 (15.2%)	
Deaths			0.001^c^
YES	4 (5.3%)	10 (30.3%)	
NO	71 (94.7%)	23 (69.7%)	

^a^Chi-square test; b, Fisher Freeman Halton test; c, Chi-square test with Yate’s correction; d, Mann Whitney test

The incidence of IVH stage II to IV was inversely proportional to multiple criteria which are the following:

- Gestational age; incidence was significantly higher in children born from 24 + 0 to 28 + 6 weeks of gestation than born from 29 + 0 to 32 + 0 weeks of gestation ((84.8% vs 15.2%); *p* = 0.00003).
- Birth weight; incidence was significantly higher in newborns with birth weight less than 1000 g (72.7% vs 27.3%); *p* = 0.00007))
- Lower Apgar score in first (6(1–10) vs 4(1–10); *p* = 0.000127) and fifth minute of life (8(2–10) vs 7(1–9); *p* = 0.00024) also had higher incidence of IVH.

Moreover, IVH developed more often in children diagnosed with intrauterine infection (78.8% vs 21.2%; *p* = 0.009). Fourteen of 108 (12.9%) patients died, including 10 (30.3%) patients with stage II to IV of IVH (*p* = 0.001). All children that died were born from 24 + 0 to 28 + 6 weeks of gestation (23.7%).

Analysis showed higher prevalence of stage II to stage IV IVH in children with the genotype TT FN1 *rs10202709* gene polymorphism (OR 7237 (1046–79.59; *p* = 0,044)). Additionally within this gene, the T allele in particular also showed a significantly higher prevalence of stage II to stage IV IVH in the infants (OR 2352 (1093–4999); *p* = 0,027).

Our investigation did not confirm any significant prevalence for IVH development in any other genotypes/alleles of FN1. Genotype and allele distribution of the polymorphisms in infants with/without IVH stage I and with IVH grade II–IV is presented in Table 3.

**Table 3:** Genotype and Allele distribution of infants

Gene symbol		Group without IVH *N* = 53(%)	Group with IVH grade I- IV *N* = 47(%)	*P* value	OR	Group without IVH and IVH I *N* = 69 (%)	Group with IVH grade II- IV *N* = 31 (%)	*P* value	OR
FN1 rs3796123	Genotype								
	AA	30 (52.63)	29 (56.86)	-	References	40 (53.33)	19 (57.58)	-	References
	AT	23 (40.35)	19 (37.25)	0,853	0,854 (0.358-2.033)	30 (40.00)	12 (36.36)	0.868	0,842 (0.320–2.163)
	TT	4 (7.02)	3 (5.88)	1000	0,776 (0.105–5.046)	5 (6.67)	2 (6.06)	1000	0.842 (0.074–5.757)
	Allele								
	A	83 (72.81)	77 (75.49)	-	References	110 (73.33)	50 (75.76)	-	References
	T	31 (27.19)	25 (24.51)	0,770	0.869 (0.449–1674)	40 (26.67)	16 (24.24)	0.845	0.880 (0.419–1790)
FN1 rs1968510	Genotype								
	GG	49 (85.96)	46 (90.20)	-	References	66 (88.00)	29 (87.88)	-	References
	GA	7 (12.28)	5 (9.80)	0.896	0.761 (0.178–3.02)	8 (10.67)	4 (12.12)	1.000	1.138 (0.232–4.663)
	AA	1 (1.75)	0 (0.00)	1.000	0 (0–42.39)	1 (1.33)	0 (0.00)	1.000	0.000 (0.000–90.1)
	Allele								
	G	105 (92.11)	97 (95.10)	-	References	140 (93.33)	62 (93.94)	-	References
	A	9 (7.89)	5 (4.90)	0.542	0.604 (0.153–2.086)	10 (6.67)	4 (6.06)	1.000	0.903 (0.199–3.286)
FN1 s10202709	Genotype								
	CC	40 (70.18)	34 (66.67)	-	References	55 (73.33)	19 (57.58)	-	References
	CT	16 (28.07)	11 (21.57)	0.814	0.809 (0.296–2.154)	18 (24.00)	9 (27.27)	0.602	1.447 (0.485–4.105)
	TT	1 (1.75)	6 (11.76)	0.101	7.059 (0.779–332.6)	2 (2.67)	5 (15.15)	0.044	7.237 (1.046–79.59)
	Allele								
	C	96 (84.21)	79 (77.45)	-	References	128 (85.33)	47 (71.21)	-	References
	T	18 (15.79)	23 (22.55)	0.275	1.553 (0.741–3.284)	22 (14.67)	19 (28.79)	0.027	2.352 (1.093–4.999)
FN1 rs6725958	Genotype								
	CC	17 (29.82)	17 (33.33)	-	References	21 (28.00)	13 (39.39)	-	References
	AC	22 (38.60)	23 (45.10)	1.000	1.045 (0.391–2.796)	30 (40.00)	15 (45.45)	0.829	0.808 (0.289–2.274)
	AA	18 (31.58)	11 (21.57)	0.481	0.611 (0.197–1.871)	24 (32.00)	5 (15.15)	0.117	0.336 (0.081–1.241)
	Allele								
	C	56 (49.12%)	57 (55.88%)	-	References	72 (48.00)	41 (62.12)	-	References
	A	58 (50.88%)	45 (44.12%)	0.392	0.762 (0.430–1.349)	78 (52.00)	25 (37.88)	0,077	0.563 (0,297–1.057)
FN1 s35343655	Genotype								
	GG	35 (61.40)	31 (60.78)	-	References	46 (61.33)	20 (60.61)	-	References
	GA	17 (29.82)	17 (33.33)	0.939	1.129 (0,454–2.805)	23 (30.67)	11 (33.33)	1.000	1.1 (0.403–2.903)
	AA	5 (8.77)	3 (5.88)	0.903	0.677 (0.098–3.833)	6 (8.00)	2 (6.06)	1.000	0.767 (0.070–4.803)
	Allele								
	G	87 (76.32)	79 (77.45)	-	References	115 (76.67)	51 (77.27)	-	References
	A	27 (23.68)	23 (22.55)	0.973	0.938 (0.472–1.855)	35 (23.33)	15 (22.73)	1.000	0.966 (0.449–2.009)

## Discussion

IVH originates from a complex etiology, with an occurrence prevalent in a relatively significant portion of preterm infants. The delicacy of the germinal matrix vasculature plays a key role in the development of this complication. Multiple factors contribute to the sensitivity of the germinal matrix vasculature and its propensity to be the source of hemorrhage in preterm infants. On a gross structural level, when compared with other regions of the brain such as the gray (GM) or white (WM) matter, the germinal matrix has a larger blood vessel cross area and vascularity [17]. In the same degree, the germinal matrix vasculature is comprised of molecular and cellular deficiencies that make it more fragile than neighboring vessels.

A proper epithelial boundary of structural congruence and coherence plays an important role in the guidance of secondary cell structures for precise anatomical positioning and its function. Compared with the surrounding brain regions (GM and WM), the germinal matrix vessels have shown a decreased coverage by astrocyte perivascular end-feet [29]. This decrease in coverage is especially evident between the 23rd and 34th week of fetal gestation.

Additionally, research from Braun and colleagues has shown that there is a decrease in density of pericytes surrounding the vessels in these particular areas of the brain with a decrease observed surrounding BBB astrocyte populations [30]. Both the astrocytes and pericytes are crucial for the integrity of the BBB [31, 32]. The observed vulnerability of certain BBB areas to the occurrence of IVH may be influenced by the populations of BBB supporting glial cells and their limited function as a result of their decreased numbers. Schreiber and colleagues argue that the breakdown of the blood brain barrier is a hallmark of unwanted blood vessel permeation into the cerebral cortex [33]. Their results highly supported a genetic predisposition to this increase in BBB permeability. The leakage is highly correlated with intraventricular hemorrhages in the germinal matrix regions due to the lack of structural integrity observed.

Furthermore, since fibronectin plays an instrumental role in the stability of vasculature, the deficiency of fibronectin in the basal lamina of the germinal matrix, compared with the other areas of the brain, may influence the integrity of vasculature structure resulting in particular weakness and greater permeability [22]. A deeper understanding of why such deficiencies occur and their link to phenotypic and genotypic expression may pave the way for more effective interventions and treatments.

The role of genetics in IVH has become clearer as the field advances [34]. From our previous studies, we were able to highlight the significance of methylenetetrahydrofolate reductase (MTHFR 1298A > C) polymorphism as a possible risk factor for IVH due to increased levels of plasma homocysteine [35]. Similarly, there was approximately a 3.5-fold increased risk of developing IVH in preterm infants with the GT eNOS *894G* > *T* polymorphism of endothelial nitric oxide synthase (eNOS), a gene associated with blood flow [12]. Prasun P et al. examined polymorphisms in VEGF and showed a higher prevalence of IVH in infants with GA/AA and AA/AC genotypes in VEGF RS1570360 and VEGF RS699947, respectively [36].

The fibronectin (FN1) gene has been assessed in relation to multiple disorders. Polymorphisms and mutations in the FN1 gene have shown to possess an association with renal glomerulopathy [37, 38], spondylometaphyseal dysplasia [39], lung fibrosis in systemic sclerosis [25], and knee osteoarthritis [40]. Its relevance has also been studied in schizophrenia [41] and some cancers [42, 43] among other observed disorders [44]. Considering the plethora of gathered data, there has yet to be any reports of FN1 gene polymorphisms in the context of IVH.

In our study, we assessed the significance of fibronectin gene polymorphism in the incidence of IVH in 108 infants born between 24 + 0 and 32 + 0 weeks of gestation. Within this distribution, 51 (47.2%) infants developed IVH and out of the 5 gene polymorphisms assessed (*rs3796123*; *rs1968510*; *rs10202709*; *rs6725958*; *rs35343655),* one genotype (TT-FN1 *rs10202709* gene) and one allele (T-FN1 *rs10202709* gene) showed a significantly higher prevalence of IVH type II to IV than the rest. Infants with the single nucleotide polymorphism TT in the *rs10202709* gene were roughly 7 times (OR 7237 (1046–79.59; *p* = 0,044)) more likely to develop IVH stage II to IV as a complication to preterm birth. In the same gene, *rs10202709*, infants with the T allele were also more than twice as likely to develop IVH stage II to IV (OR 2352 (1093-4999); p = 0,027). The presented data suggests a possible relationship between the FN1 rs10202709 polymorphisms and IVH occurrence.

To date, only a few studies have been published in regard to the FN1 rs10202709 polymorphism. An insufficiency of information is apparent with regard to the phenotype result of this single nucleotide polymorphism. Murat and colleagues assessed the FN1 polymorphism site rs10202709 as a possible factor in developing calcium oxalate stones in the Uighur population but obtained no significant correlation [26]. The FN1 rs10202709 was also assessed by Yung et al. in relation to osteoarthritis in a Han Chinese population; however, out of the six polymorphisms (rs10202709, rs6725958, rs940739, rs2304573, rs11651, and rs3796123), it was concluded that the rs940739 A/T was the only one with a genetic association to osteoarthritis [40]. Research groups attempted to provide an adequate explanation behind the function and molecular architecture with respect to FN1 protein post polymorphic changes [26, 40, 45]. However, regardless of which particular polymorphic change had occurred, a significant disruption in the FN1 protein was observed. The site of the polymorphic change within each study may be a result of the location of the FN1 protein affected. A different effect based on location may be observed according to tissue classification of the FN1 protein. Despite this difference in tissue locality for each significant polymorphic change on the FN1 protein, the resulting effect produces inappropriate bond strength and incorrect fibronectin conformation causing inadequate function for proper physiological utilization. This in turn is very likely to have occurred in the FN1 rs10202709 polymorphisms presented in this study.

The results from our study indicate, for the first time (to the authors’ knowledge), that a possible fibronectin polymorphism has the potential to significantly predispose preterm infants to IVH. Fibronectin is a crucial component for the proper vascular stability. As such, these findings may further explain the tendency of why certain babies are more prone to the development of a hemorrhage in the germinal matrix and as a result IVH. The obtained results of the rs10202709 polymorphisms relationship provide evidence for the importance of future investigation, with larger sample sizes, in order to fully understand the association between the FN1 polymorphism rs10202709 and its phenotypic consequences on IVH.

## Electronic supplementary material

ESM 1 (XLS 30 kb)
